# An exploratory in-situ dynamic mechanical analysis on the shearing stress–strain mechanism of human plantar soft tissue

**DOI:** 10.1038/s41598-024-62713-9

**Published:** 2024-05-25

**Authors:** Ran Huang, Xinyi Ning, Longyan Wu, Jun Zhu, Lisheng Tang, Xin Ma

**Affiliations:** 1 Academy for Engineering and Technology, Fudan University, Shanghai, 200433 China; 2 Yiwu Research Institute of Fudan University, Yiwu, 322000 Zhejiang China; 3 Center for Innovation and Entrepreneurship, Taizhou Institute of Zhejiang University, Taizhou, 318000 Zhejiang China; 4 Shanghai Sixth People’s Hospital, Shanghai Jiao Tong University, Shanghai, 200233 China

**Keywords:** Plantar soft tissue, In-vivo/in-situ, Shear modulus, Dynamic mechanical analysis, Biomechanical characterization, Biophysics, Biomedical engineering, Biological physics

## Abstract

A DMA (dynamic mechanical analysis)-like device based on the principle of classical viscoelasticity testing is invented to investigate the in-situ/in-vivo shear-bearing mechanism of plantar soft tissue. Forty-three volunteers were recruited for the shear-strain test in the longitudinal and transverse directions at five anatomical spots on the plantar surface. Several encouraging observations indicated significant variances among different spots and individuals, implying that the outer forefoot surrounding the second, fifth metatarsal head is a more intensive shear-bearing region on the plantar surface compared to the inner forefoot under the first metatarsal head, and drawing the hypothesis of a significant effect of BMI on the shear-bearing property. The speculations agree with our expectations and other previous research. The feasibility and practical value of this novel approach are substantiated, and these intriguing discoveries provide foundational underpinnings for further in-depth investigations.

## Introduction

The soft tissue of the human plantar bears significant weight and loads in daily activities, playing a crucial role in absorbing and dispersing impact forces during motion^1–3^. Intensive physical activity^4^, imbalanced muscular strength^5,6^, aging^7^, diabetes^8^, and other factors can lead to the degeneration, injury, and even ulceration of the soft tissues on plantar, which can significantly hinder daily functioning^9^. Therefore, the investigation on the material characteristics of human plantar soft tissue is critical in the management of plantar diseases and in developing bionic materials for the production of prosthetics^10,11^.

A number of researches attest that the shear-bearing property of the human plantar soft tissue allows it to adapt to the shape and hardness of different ground, provide stable support, and help maintaining the body balance, therefore plays a significant influence^12–14^. The investigation of the shear-bearing capacity of plantar tissue materials is a topic of great importance in the fields of sports biomedicine and diabetic footcare. This research is crucial for designing and developing functional insoles, adaptive outsoles, and other assistive devices, optimizing athletic performance, and preventing sports-related injuries and ulceration^15^. The shear-bearing properties of plantar soft tissues have also constituted a perennially critical area of research within the realms of clinical medicine and biomedical engineering^16^.

Subsequently, the measurement of shearing force on plantar surface has naturally become a hot topic in the field, a number of techniques and methods have been developed for the general foot during active loading or walking^16–18^. Nevertheless, the anti-shearing material property, usually characterized by modulus, of plantar tissue, which can be viewed as a cushion elastomer in the classical material science, has been very less reported. Obviously, the physiological structure of the plantar soft tissue is complex, and due to the strict requirement of non-invasive testing, the current assessment of the material and mechanical properties of plantar soft tissue are merely conducted on cadaver specimens. However, the characterizations obtained under postmortem condition are controversial to reflect the reliable physiological properties of the living body in reality^19^. Up to present, measurement of shear or frictional forces is considered to be important but yet routine clinical practice, due to a series of technical challenges^20^.

To address the aforementioned issues, we have innovatively designed a Dynamic Mechanical Analysis (DMA)-like test instrument based on the classical testing method of viscoelastic materials, which creatively enables in-vivo/in-situ periodical stress–strain test on human plantar soft tissue. The mechanical principles of this testing equipment are introduced in detail in Ref.^21^. With the assistance of finite element analysis (FEA) simulation, the feasibility of our instrument and methodology has been substantiated^22^. Building upon previous works, this manuscript reports the results of shear stress–strain tests on five key spots on the plantar surface (beneath the first metatarsal, second metatarsal, fifth metatarsal heads, the edge of the lateral arch, and the center of the heel) for 43 volunteer subjects. Within the course of the tests, we discovered several intriguing findings, and the inferences drawn from them are elaborated in the following sections.

## Methods

### Human ethical statements

We confirm that all methods were carried out in accordance with relevant guidelines and regulations. We confirm that all experimental protocols were approved by The Ethical Review Committee of Huashan Hospital, Fudan University (HIRB). We confirm that informed consent was obtained from all subjects and/or their legal guardian(s) in the written mode (see the Related File 1).

### Instrument and experiment procedure

**Figure 1:**
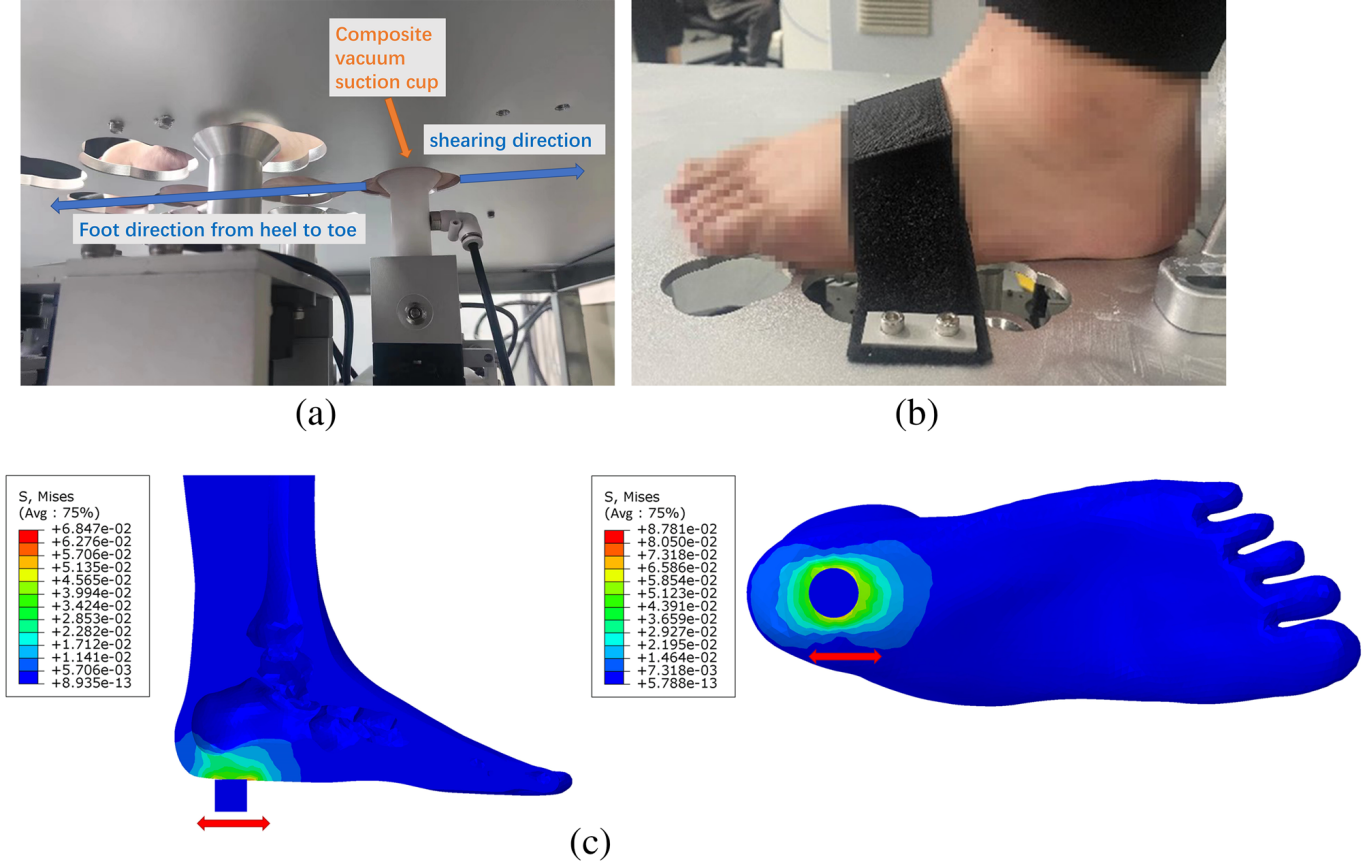
The demonstration of test posture, principle and mechanism: (**a**) the test setup from undercover view (**b**) the test setup of volunteer’s foot (the resolution of foot is downgraded for privacy protection); (**c**) a FEA sectional illustration of the shearing test. (The subfigure (**a**) and (**b**) have been publicly disclosed, and copyrights for re-use are authorized in this paper.).

**Figure 2:**
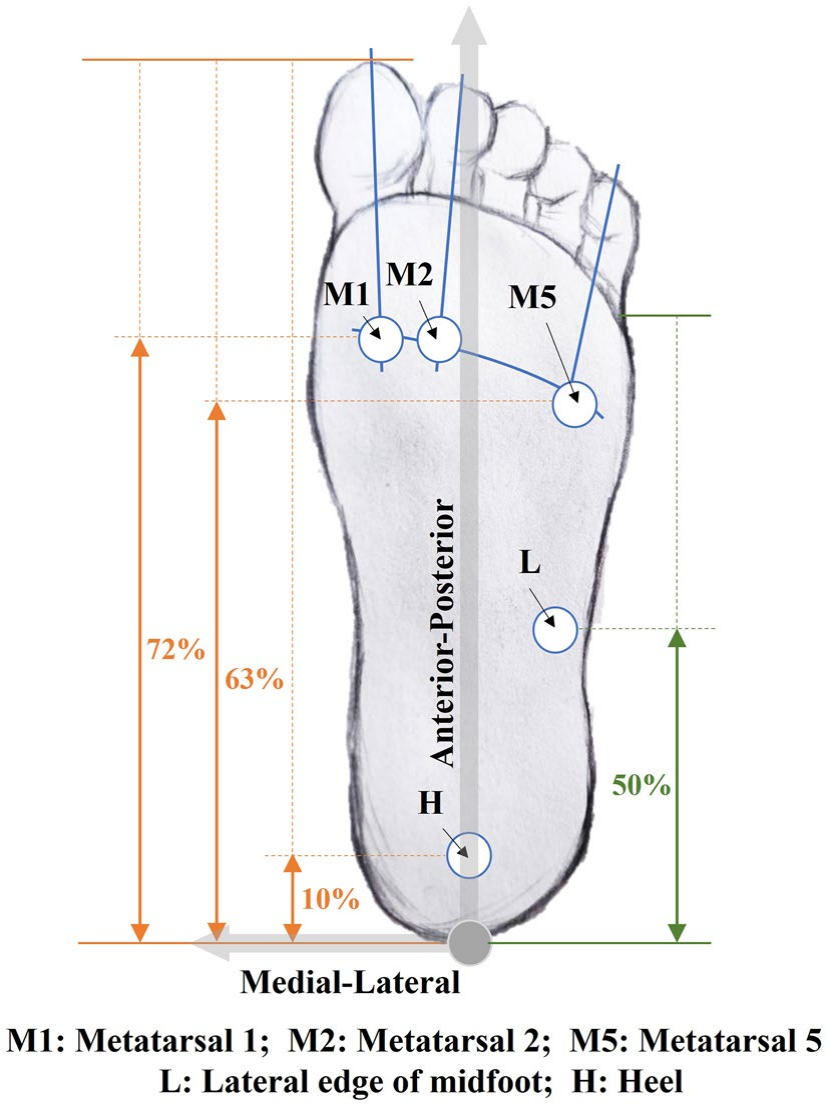
Illustration of the locations of the five test points on the plantar of interest (beneath the first metatarsal (M1), second metatarsal (M2), and fifth metatarsal (M5) heads, lateral edge of foot arch (L), and directly beneath the center of the heel (H)). Note that the 50% on the right to determine L is colored differently as it is based on a different 100% norm. (The figure has been publicly disclosed, and copyrights for re-use are authorized in this paper.).

The theoretical framework and design of our devised testing apparatus have been extensively described in our previous work^21–23^. The device is capable of executing three distinct types of stress–strain measurements: tension and compression, shear and torsion. However, this work only focuses on the shearing test, since the feature of anti-shearing stability of plantar soft tissue is the priori property that we are interested in. The test posture, principle and mechanism of test are illustrated in Fig. 1. The schematic diagram of the locations of the five test spots, which are considered to be critical in load-bearing or sensitive to ulceration, on the plantar is shown in Fig. 2. Each spot was subjected to shearing in the longitudinal (from heel to toe) and transverse direction.

Test site locations are chosen based on a number of references, as there are common concerns among studies on the importance of particular places for plantar functioning. The heel, the fifth, and the first metatarsal heads are tested at about 10%, 63%, and 72% of the foot length measured from the posterior-most point in the anterior–posterior direction, respectively, in reference to earlier research on foot morphology^24^ and plantar pressure^25^. Based on the pressure distribution on the plantar^26–28^, the line M1-M2 is seen as perpendicular to the longitudinal direction, with both situated at the intersection of the 72% “meridian” and the axis of the first and second metatarsal bone. Similarly, M5 is situated at the intersection of the 63% "meridian" and the axis of the fifth metatarsal bone. Point L is located at the midpoint of the outer lateral of the arch. Note that the 50% on the right in Fig. 2 is based on a different 100% norm.

During the test, the subject's foot is fixed with straps, and the point for measurement is aligned with the testing probe, and adjoined by a composite vacuum suction cup^22,23^. The contact area between skin and suction cup is 380 mm^2^ (*D* = 22 mm). The detector performing periodic reciprocating motion is driven by a sine slider mechanism. The sampling frequency is 1000 Hz, i.e., the sensors collect 1000 points per second. Each test is repeated three times. In each trial, the shear reciprocating motion lasts for 10 s, with a setup amplitude of ± 2 mm and frequency of 2 Hz. The parameters were determined based on prior knowledge and observations during the experiment. The subjects did not experience any discomfort or abnormal feelings during all experiments. It is important to note that the displacement in reality is not exactly the setup value (± 2 mm), due to the resistance of tissue in real test, the total displacement is observed unignorably smaller than 4 mm, therefore the strain is measured by independently installed sensor in the device to calculate the moduli.

### Participants

We recruited a total of 43 volunteer participants, 22 males (age: 27.91 ± 7.10 years, height: 175.86 ± 5.60 cm, body mass: 76.05 ± 16.00 kg) and 21 females (age: 30.57 ± 7.09 years, height: 162.90 ± 5.83 cm, body mass: 53.10 ± 6.27 kg). All subjects were required to have had no trauma to their feet in the past 12 months before data collection, and the plantar shall be observed to be in a healthy state, that appears not to have any injuries, infections, or pain. The assessment is done by researchers who have been trained by professional clinician in our group. Prior to testing, the researcher explained the procedure and purpose of the study to the subject, collected personal and clinical information such as age, height, weight, foot length, foot width, est. average steps per day, and exercise habits from the subjects, which was subsequently anonymized during the data processing phase. For the self-estimation on exercise intensity, according the WHO guide^29^, the benchmark is set as: LOW: less than 100 min of moderate aerobic activity per week; MIDDLE: 100–200 min of moderate or 50–100 min of vigorous aerobic activity per week; HIGH: > 200 min of moderate or > 100 min of vigorous aerobic activity per week. And the moderate intensity refers to 60–75% of maximum heart rate (HRmax), vigorous refers to > 75% HRmax, with the formula for estimating maximum heart rate as HRmax = 207–0.7*age. All subjects voluntarily signed the testing protocol agreement (Related Files 1) before testing commenced. The Ethical Review Committee of Huashan Hospital, Fudan University (HIRB) approves the ethical review (Related Files 2).

For the two non-structured indicators: gender and exercise habits, we employed a structured numerical feature encoding approach. For the gender, a factorized encoding method was applied, representing males as 1 and females as 2. For exercise habits, we assigned values to indicate an ordered relationship: 0 for low intensity, 5 for middle intensity, and 10 for high intensity. Furthermore, prior to calculating correlation coefficients, the data underwent normalization by scaling it to a range between 0 and 1 through a min–max normalization operation.

### FEA simulation test

The FEA simulation test was conducted on a finite element full foot model with soft tissue and calcaneus bone. The machine is directly constructed with the original design drawing, and the material properties of the heel pad are set to approximate plantar soft tissue referred with previous report^22^. The simulation sketch is shown in Fig. 1c. The suction cup is simplified as a rigid cylinder with D = 22 mm, with a binding constraint to the heel, the reciprocal shearing motion is processed with a sinusoidal curve of ± 2 mm amplitude at a frequency of 2 Hz.

### The definition of *modulus*

**Figure 3:**
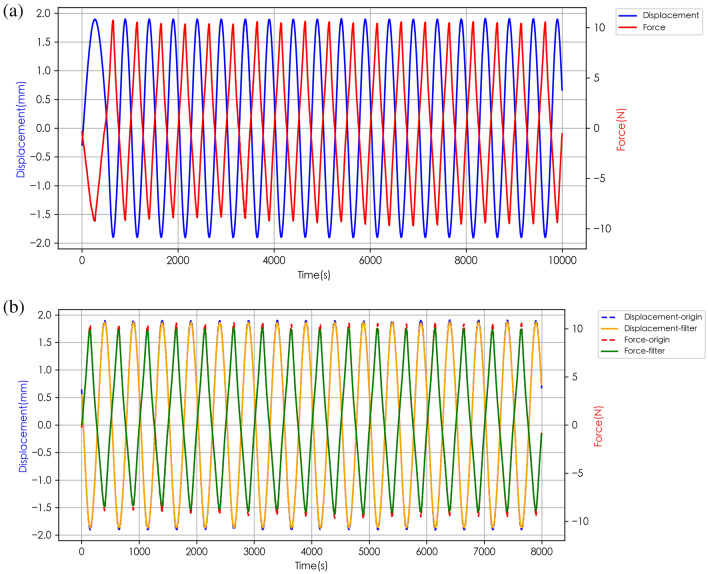
Force–displacement curve of subject No.4 (heel): (**a**) original data; (**b**) comparison of the original data and the Gaussian filtering results.

The original data obtained from the device are periodical force and displacement, as illustrated in Fig. 3 in the following section. The entire amplitude from peak to valley is counted as force in Newtons. The results are obtained by averaging the values of multiple tests after filtering. Then the new shear modulus is calculated as

1 $$\begin{array}{c}{H}_{S}=\frac{{F}_{S}/A}{\Delta x/L}\end{array}$$

where *F*_S_ is the combined force of shearing of two directions of the oscillation, and note the Δ*x* is also the displacement of tissue oscillated to both sides. The contact area A is 380 mm^2^. The *L* is a nominal “original size”, which is set to be 10 mm. Although the “true” original size is a complicated and challenging issue in the living organ measurement, by considering that the shape and size are also part of the “eigen” tissue properties to be reflected by the stress–strain behavior, the definition of this “apparent modulus” can satisfy our current need to measure and examine the plantar tissue as a healthy indicator, for the detailed discussion on the modulus definition, please refer to ref.^22^.

## Results and discussion

The newly-invented instrument is testified to provide precious and consistent results. As an example, Fig. 3a shows the periodic stress–strain behavior presented by the test on point (H) of subject 4. The direction of the displacement and force is reversed to more intuitively and clearly display the peaks and valleys of the signal, and the cross of two curves at zero, which is taken as an important criterion on the accuracy of the device. During the actual test process, the oscillation of the shear reciprocating motion gradually increased from a lower frequency, and it took about 1 s to reach the default frequency and continue to output stably. Therefore, we select the data after t = 2 s in the post-processing stage for calculation and further analysis of the average force.

The Fig. 3 clearly shows that the force and displacement curves intersect at zero, and the waveform output is accompanied by jitter. These are attributed to noise interference on the sensor signal during the testing process. We applied the first-order Gaussian filtering to process the noise fluctuation, with the standard deviation setting as 10 for the Gaussian kernel. The contrast before and after filtering is shown in Fig. 3b. One thing to note that, in Fig. 3 the upper limit of the force curve reaches around 10 N, while the lower limit is obviously lower than 10 N. This is because the “appear moduli” on the two oscillation directions can be different even on one spot, due to the heterogeneity of the tissue’s geometry and structure, and this is easier to understand by imaging the transverse shearing on the lateral arch (spot L). Meanwhile, the displacements in both directions are all close to 2 mm because that is fixed by the mechanism. This is also one of the reasons we define the modulus with combined force and displacement in Eq. (1), to simplify the treatment of different moduli in one shearing mode.

The calculated modulus for 43 volunteers with their basic personal and clinical information are provided in Table 1. (Full data including original force and displacement can be referred to Table S1) Each modulus is the average of ~ 20 cycles of measurements in three times of tests. Due to the privacy protection, the personal and clinical info are reported in intervals. Data with full details, and experimental results of a single test are available upon request to the authors with conditional permission and policy.

**Table 1:** The clinical info and shearing moduli of living tests.

Subject index	Gender	Age	Height (cm)	Weight (kg)	BMI	Foot length (mm)	Foot width (mm)	Steps/daily avg	Exercise Intensity	H_LS__M1 (MPa)	H_LS__M2 (MPa)	H_LS_ _M5 (MPa)	H_LS__L (MPa)	H_LS__H (MPa)	H_LS__AVG (MPa)	H_TS__M1 (MPa)	H_TS__M2 (MPa)	H_TS__M5 (MPa)	H_TS__L (MPa)	H_TS__H (MPa)	H_TS__AVG (MPa)
1	M	36–40	186–190	126–130	35–37	276–280	96–100	5000	Low	0.1209	0.1509	0.1840	0.2655	0.2168	0.1876	0.1045	0.1237	0.1509	0.1504	0.1725	0.1404
2	F	31–35	156–160	56–60	21–23	221–225	81–85	3000	Low	0.0632	0.1047	0.1276	0.1648	0.1522	0.1225	0.0631	0.0872	0.0748	0.0902	0.0674	0.0766
3	F	26–30	156–160	36–40	15–17	216–220	76–80	4500	Low	0.0876	0.0914	0.0942	0.1532	0.1664	0.1185	0.0687	0.0664	0.0625	0.0844	0.0990	0.0762
4	F	31–35	166–170	56–60	20–22	236–240	76–80	5000	Middle	0.1400	0.1281	0.1146	0.1665	0.1578	0.1414	0.1110	0.1264	0.0603	0.1041	0.1252	0.1054
5	M	31–35	166–170	81–85	27–29	246–250	91–95	5000	High	0.1166	0.1043	0.1093	0.1520	0.1661	0.1297	0.0876	0.0689	0.0826	0.0980	0.1350	0.0944
6	M	31–35	171–175	76–80	25–27	251–255	86–90	5000	Middle	0.1113	0.1164	0.1314	0.2265	0.1999	0.1571	0.1102	0.0760	0.0879	0.1361	0.1157	0.1052
7	F	21–25	171–175	51–55	16–18	236–240	76–80	4500	Low	0.0933	0.0661	0.0853	0.1306	0.1847	0.1120	0.0567	0.0473	0.0599	0.0626	0.1391	0.0731
8	F	21–25	171–175	56–60	18–20	236–240	81–85	9000	Middle	0.0716	0.0707	0.0640	0.1464	0.1624	0.1030	0.0589	0.0569	0.0427	0.0717	0.1413	0.0743
9	M	36–40	171–175	81–85	26–28	241–245	86–90	5500	Low	0.0806	0.1142	0.1301	0.2284	0.1491	0.1405	0.0782	0.0946	0.0929	0.1478	0.0976	0.1022
10	F	26–30	166–170	56–60	18–20	236–240	76–80	6000	Low	0.0711	0.0901	0.1055	0.1338	0.2670	0.1335	0.0559	0.0694	0.0884	0.0844	0.2327	0.1061
11	M	21–25	171–175	71–75	23–25	241–245	86–90	8000	Low	0.0611	0.0972	0.1059	0.1446	0.1607	0.1139	0.0617	0.1027	0.0958	0.0874	0.1100	0.0915
12	F	21–25	156–160	41–45	16–18	211–215	81–85	8000	Low	0.1106	0.0653	0.0744	0.1072	0.1570	0.1029	0.0650	0.0525	0.0615	0.0617	0.0654	0.0612
13	M	26–30	171–175	76–80	24–26	256–260	96–100	8000	Middle	0.1161	0.1566	0.1449	0.1832	0.1960	0.1594	0.0963	0.1327	0.0898	0.1155	0.1302	0.1129
14	F	36–40	161–165	51–55	18–20	221–225	76–80	7000	Middle	0.0994	0.1024	0.0936	0.1505	0.0984	0.1089	0.0664	0.0721	0.0544	0.0748	0.0584	0.0652
15	M	21–25	176–180	71–75	22–24	251–255	86–90	5000	Low	0.0937	0.1514	0.1146	0.1189	0.1285	0.1214	0.1005	0.0897	0.0984	0.0769	0.0794	0.0890
16	F	46–50	161–165	56–60	21–23	216–220	76–80	6000	Middle	0.0911	0.1521	0.1463	0.1747	0.1119	0.1352	0.0704	0.1080	0.0784	0.1058	0.0845	0.0894
17	F	21–25	156–160	41–45	17–19	221–225	91–95	10,000	Low	0.0808	0.0710	0.0839	0.1912	0.1623	0.1178	0.0709	0.0808	0.0619	0.1056	0.0708	0.0780
18	M	21–25	181–185	56–60	16–18	271–275	81–85	4000	Middle	0.0601	0.0719	0.0779	0.0820	0.1104	0.0805	0.0408	0.0675	0.0606	0.0403	0.0745	0.0567
19	M	21–25	171–175	76–80	25–27	241–245	81–85	8000	Middle	0.0880	0.1220	0.1583	0.1474	0.2137	0.1459	0.0829	0.0971	0.1128	0.0787	0.1403	0.1024
20	M	21–25	166–170	71–75	24–26	231–235	81–85	8000	High	0.1344	0.1090	0.1590	0.1642	0.1935	0.1520	0.1105	0.0908	0.1085	0.0776	0.1308	0.1036
21	M	21–25	176–180	81–85	25–27	251–255	91–95	8000	Middle	0.1132	0.1266	0.1407	0.2124	0.1735	0.1533	0.1057	0.1057	0.0883	0.0963	0.1350	0.1062
22	M	26–30	171–175	76–80	24–26	241–245	81–85	7000	Low	0.1345	0.1322	0.1735	0.1956	0.1895	0.1651	0.1223	0.1062	0.1013	0.1188	0.1387	0.1175
23	M	36–40	166–170	51–55	16–18	241–245	81–85	7000	Low	0.0502	0.0672	0.0989	0.1300	0.1787	0.1050	0.0537	0.0595	0.0753	0.0544	0.0691	0.0624
24	M	26–30	171–175	86–90	28–30	246–250	81–85	10,000	Middle	0.1043	0.1190	0.1476	0.1687	0.1955	0.1470	0.0998	0.0953	0.0887	0.0773	0.1755	0.1073
25	F	26–30	156–160	51–55	20–22	236–240	76–80	7000	Middle	0.0555	0.0969	0.0652	0.1179	0.0904	0.0852	0.0554	0.0375	0.0772	0.0433	0.0432	0.0513
26	F	26–30	171–175	56–60	19–21	241–245	81–85	10,000	Middle	0.1152	0.1063	0.1457	0.1794	0.1929	0.1479	0.0849	0.0805	0.0625	0.0758	0.1226	0.0853
27	M	21–25	171–175	61–65	19–21	246–250	86–90	6000	Middle	0.0807	0.1072	0.0880	0.1593	0.1303	0.1131	0.0739	0.0945	0.0659	0.0607	0.0858	0.0761
28	M	21–25	181–185	96–100	27–29	271–275	91–95	6000	Low	0.1013	0.1305	0.1563	0.1508	0.1600	0.1398	0.1223	0.1291	0.1199	0.1085	0.1360	0.1232
29	F	26–30	166–170	56–60	19–21	226–230	81–85	4000	Middle	0.0604	0.1000	0.0924	0.1083	0.1069	0.0936	0.0440	0.0563	0.0636	0.0458	0.0439	0.0507
30	M	21–25	181–185	81–85	23–25	261–265	91–95	6000	Low	0.1055	0.0992	0.1044	0.1805	0.1598	0.1299	0.0976	0.0807	0.0842	0.0895	0.0917	0.0887
31	F	21–25	171–175	56–60	18–20	236–240	91–95	6000	Low	0.0809	0.1083	0.1043	0.1512	0.1704	0.1230	0.0649	0.0833	0.0688	0.0689	0.0887	0.0749
32	M	21–25	176–180	66–70	20–22	261–265	86–90	8000	Low	0.0782	0.0861	0.0742	0.1245	0.2200	0.1166	0.0590	0.0795	0.0555	0.0588	0.1036	0.0713
33	F	36–40	161–165	66–70	24–26	226–230	81–85	7000	Low	0.0882	0.1436	0.1494	0.2066	0.1427	0.1461	0.0622	0.1026	0.0917	0.1044	0.0811	0.0884
34	F	26–30	161–165	46–50	17–19	236–240	76–80	6000	Low	0.0597	0.0759	0.0819	0.0872	0.1858	0.0981	0.0330	0.0372	0.0424	0.0601	0.0750	0.0496
35	M	21–25	176–180	51–55	16–18	251–255	81–85	4000	Low	0.0644	0.0830	0.0954	0.1749	0.1908	0.1217	0.0406	0.0650	0.0465	0.0685	0.1006	0.0642
36	F	41–45	156–160	56–60	21–23	231–235	81–85	5500	Low	0.1045	0.0721	0.0918	0.1020	0.0939	0.0929	0.0545	0.0706	0.0740	0.0493	0.0652	0.0627
37	F	31–35	161–165	46–50	18–20	211–215	76–80	6000	Low	0.0787	0.0696	0.0987	0.1344	0.1825	0.1128	0.0512	0.0462	0.0785	0.0596	0.0920	0.0655
38	M	21–25	176–180	66–70	20–22	241–245	86–90	8000	Middle	0.1021	0.1147	0.0962	0.1811	0.1557	0.1300	0.0909	0.0980	0.0657	0.0784	0.1125	0.0891
39	F	36–40	151–155	46–50	18–20	221–225	76–80	6000	Low	0.1072	0.1262	0.0869	0.1283	0.1281	0.1154	0.0855	0.0734	0.0649	0.0633	0.0672	0.0709
40	F	21–25	156–160	46–50	18–20	221–225	76–80	6000	Low	0.0627	0.1433	0.1518	0.1700	0.1336	0.1323	0.0463	0.0731	0.0826	0.0605	0.0730	0.0671
41	M	21–25	171–175	61–65	19–21	246–250	81–85	8000	Low	0.0805	0.1525	0.1598	0.1694	0.1474	0.1419	0.0767	0.0915	0.0839	0.0548	0.0823	0.0778
42	M	46–50	161–165	61–65	21–23	221–225	81–85	4000	Low	0.1064	0.1236	0.1476	0.1469	0.1387	0.1326	0.0932	0.0854	0.0985	0.0838	0.0936	0.0909
43	F	31–35	156–160	46–50	18–20	221–225	76–80	8000	Middle	0.1208	0.1043	0.1425	0.1498	0.0956	0.1226	0.0879	0.0596	0.0860	0.0765	0.0493	0.0719
Average	–	29.2093	169.5349	64.8372	22.2980	240.7674	84.2674	6465.1163	–	0.0918	0.1075	0.1162	0.1572	0.1609	0.1267	0.0759	0.0819	0.0789	0.0817	0.1022	0.0841
Standard deviation	–	7.3015	8.7406	16.9873	4.1092	16.1582	5.8088	1743.6803	–	0.0234	0.8474	0.0320	0.0378	0.0382	0.0225	0.0235	0.0237	0.0215	0.0264	0.0387	0.0210
Range		29.0000	36.0000	90.0000	19.5745	62.0000	21.0000	7000.0000		0.0898	3.7895	0.1200	0.1835	0.1766	0.1071	0.0893	0.0955	0.1085	0.1100	0.1895	0.0908
FEA foot	–	–	–	–	–	–	–	–	–	0.2099	0.2135	0.1875	0.1753	0.1689	0.1910	0.1548	0.1973	0.1525	0.1377	0.1355	0.1556

For privacy protection, the personal and clinical info are reported in intervals. Data with full details, and experimental results of a single test are available upon request to the authors with conditional permission and policy.

**Figure 4:**
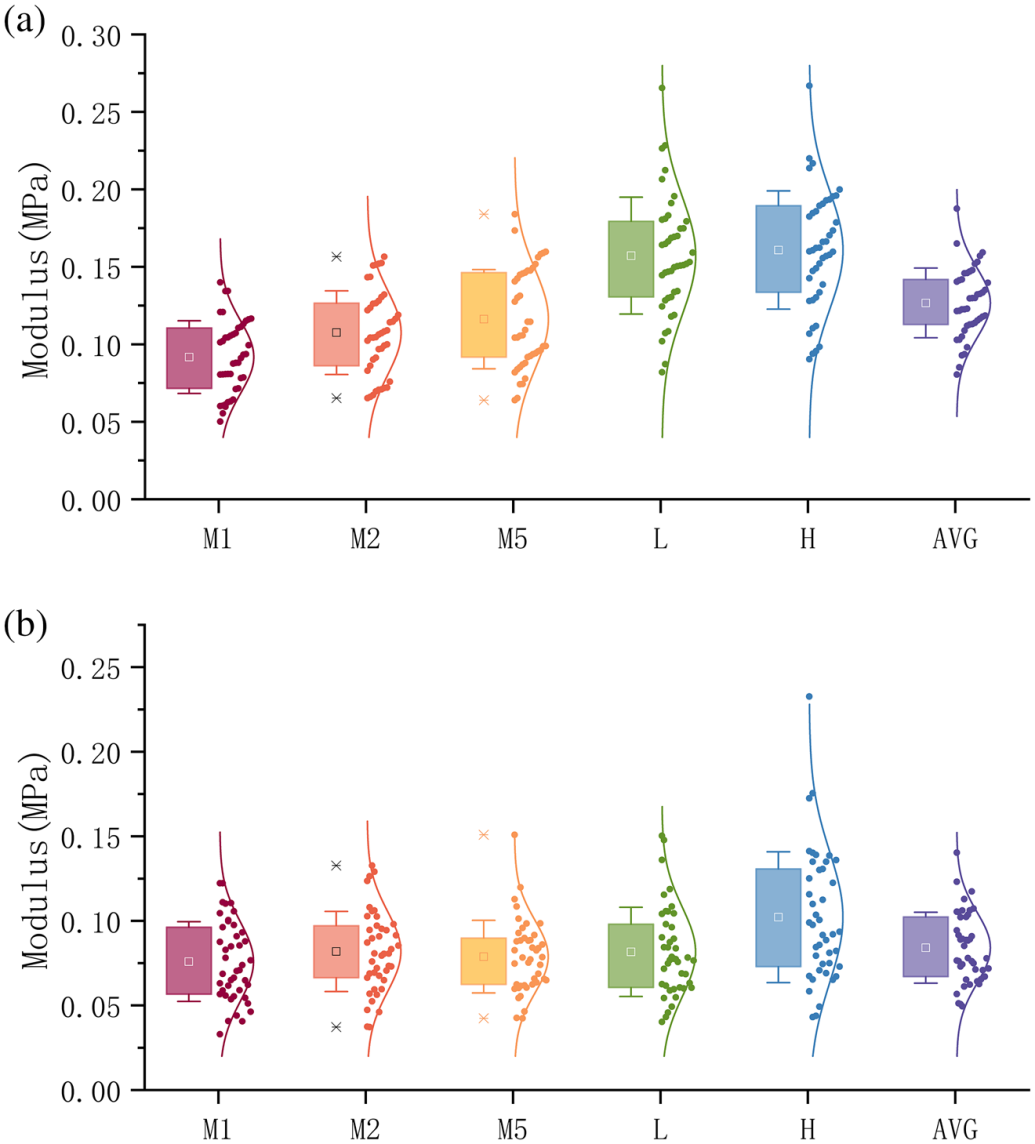
The distribution and average of modulus along various spots from M1 to L: (**a**) The results of longitudinal shearing; (**b**) The results of transverse shearing. The box is percentage of 25–75, the bar is standard deviation, the detailed distributions of data point are presented aside, with the reference of bell curve.

The first observation on Table 1 is the considerable differences in the stress modulus among the various test spots on a single subject, and among individuals, indicating notable variations in the material property of human plantar soft tissue with geometric and individual differences, and perhaps reflecting the different load-bearing capabilities during daily activities. Figure 4 (a: longitudinal, b: transverse) visualizes the distribution and average of respective modulus along various spots from M1 to L, and the detailed distribution of data point are presented along, with the reference of bell curve. The data can be observed that roughly obeys the normal distribution with acceptable exception, considering that the dataset of 43 is relatively small. We conducted Shapiro–Wilk tests on the moduli of five positions, indicating that the data for longitudinal H (p = 0.49557), L (p = 0.63921), M1 (p = 0.28936), and M2 (p = 0.07906) follow normal distributions, while the M5 (p = 0.04129) does not; and for transverse M1 (p = 0.46268), M2 (p = 0.72477), M5 (p = 0.06669) follow normal distributions, while H (p = 0.01571), and L (p = 0.03339) do not.

It can be seen in Fig. 4, the difference between the shear stress under the metatarsal bone (M1, M2, M5) are relatively small, which is reasonable since these three spots are close and together represent the forefoot area. Meanwhile, the moduli at the spot L and H are significantly greater, agreeing with the common sense that these two spots are distanced from the forefoot, and solo bear the body load and shearing stress at midfoot and heel respectively. While previous studies reported that ulcers occur more often within the forefoot^30,31^, we may hypothesize that the modulus at the spot H is greater than others, indicating that the material is stiffer and/or the fat pad is thicker here, leading a better performance in load absorption, and explaining why plantar ulcers typically occur in the forefoot rather than the heel^32^. In another word, when “the maximum shear stress appears in the middle metatarsal and heel areas”^33^, the heel bears “better” than the forefoot.

However, in similar geometric conditions, the spots on forefoot tissue can be parallelly compared, the modulus of M2 (0.1075 MPa) and M5 (0.1162 MPa) in longitudinal are higher than the M1 (0.0918 MPa), i.e., these two spots might be more intensive shear-bearing region on the forefoot. This is consistent with a previous report on the shear material properties of the cadaveric specimens of plantar soft tissue, in which the initial, middle and final modulus under M1 is respectively lower than M5 (95.9 kPa, 20.9 kPa and 38.0 kPa vs. 107.0 kPa, 29.0 kPa, and 43.8 kPa)^30^. And according to our daily experiences that most shearing happens at the upper outer edge of foot during normal locomotion^34,35^. Subsequently, several inquiries can be posed in relation to this discovery. Given the assumption that diabetic ulcers are strongly associated with plantar shear stress^36–41^, is the outer edge of forefoot the particular location more susceptible to ulcers, and should it be given greater attention in future clinical practice?

In the above speculation and analysis, we actually made an underlying hypothesis that needs to be clearly restated: we assumed that the shear modulus of tissue correlates to the exercise intensity it undergoes in daily activities. Specifically, we proposed that the tissue can be developed to be stronger and exhibits improved material properties when subjected to more intensive shear forces during daily exercise. This hypothesis can be supported by the smaller shear modulus in transverse direction at each site respectively. Nevertheless, the correlation certainly does not imply causality vice versa, i.e., high shear modulus is unnecessarily led by extensive shear-bearing. It is uncertain whether the heel has a higher shear modulus due to the fact that it experiences more intense shearing force during walking (this has also been claimed in Ref.^33^), or if it is simply because the heel tissue is thicker and stronger in all dimensions, as it directly bears the load under the axis of the entire bodyweight.

Though our present exploratory work is still far away from conclusive, we consider this topic to be intriguing and a valuable subject for future research. Furthermore, it is suggested that callus formation of the 1st and 2nd metatarsal head were related to high shear stress time integral/normal stress time integral rather than the single variables of normal stress and shear stress^34^, therefore exploring the combination of normal stress and shear stress would also be an important subject in the following studies.

To further investigate the influencing factors of the difference in results among different subjects, we comprehensively analyzed the Spearman correlation coefficients between the average modulus at different testing positions of the plantar and factors such as gender, height, weight, foot length, foot width, exercise habits, average daily walking steps, etc., with the structural processing of non-structural indicators and the data normalization.

**Figure 5:**
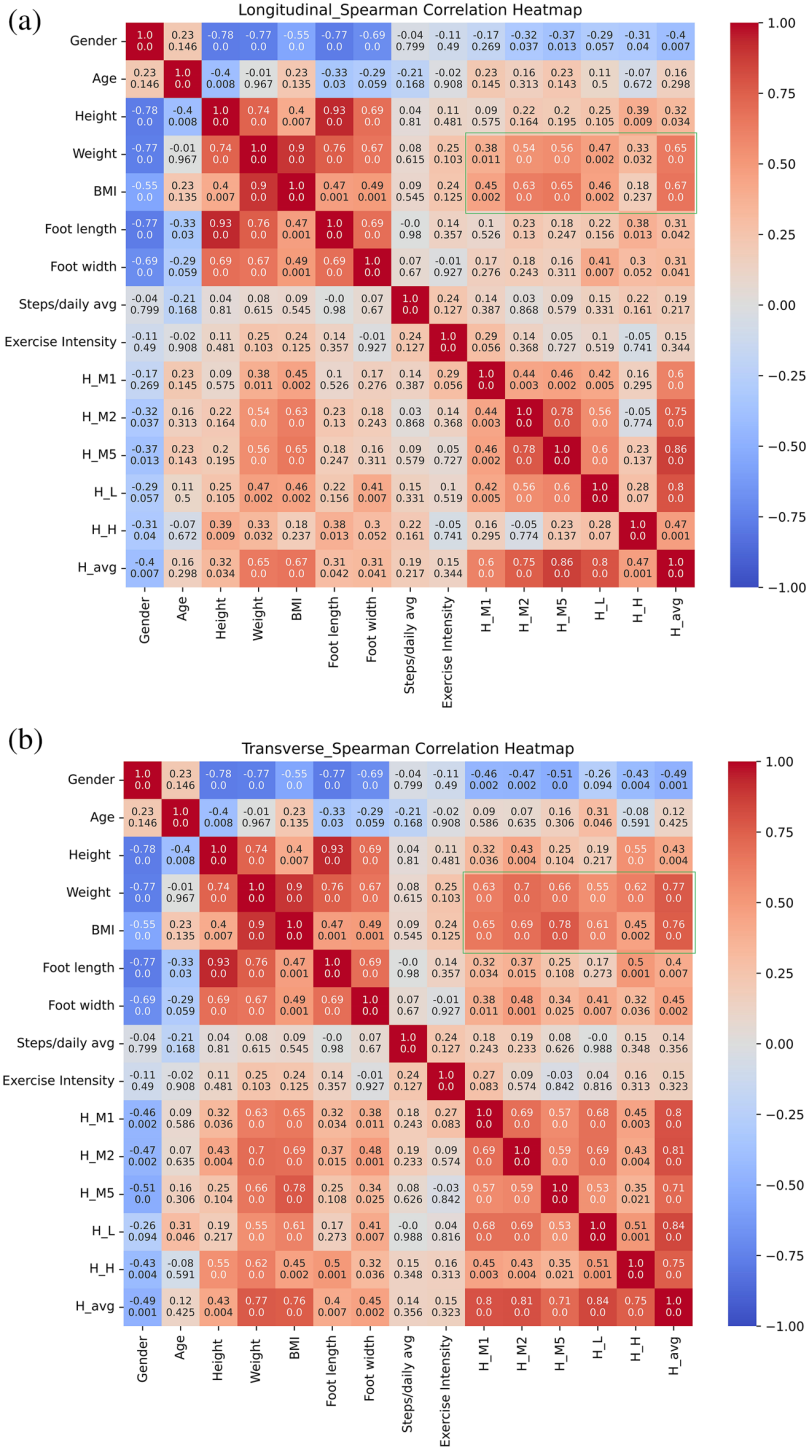
Heatmap of Spearman correlation coefficient along with P-value between shear stress modulus and various influencing factors: (**a**) The longitudinal shearing; (**b**) The transverse shearing. The “Steps/daily avg” is the average daily steps estimated by the subjects, and “Exercise Intensity” is the subjects' own evaluation, divided into three levels: low, medium, and high); Area imprinted by two frames are interested areas we focus on: the inner correlations among various plantar area (blue); and the correlation to bodyweight and BMI (green).

**Figure 6:**
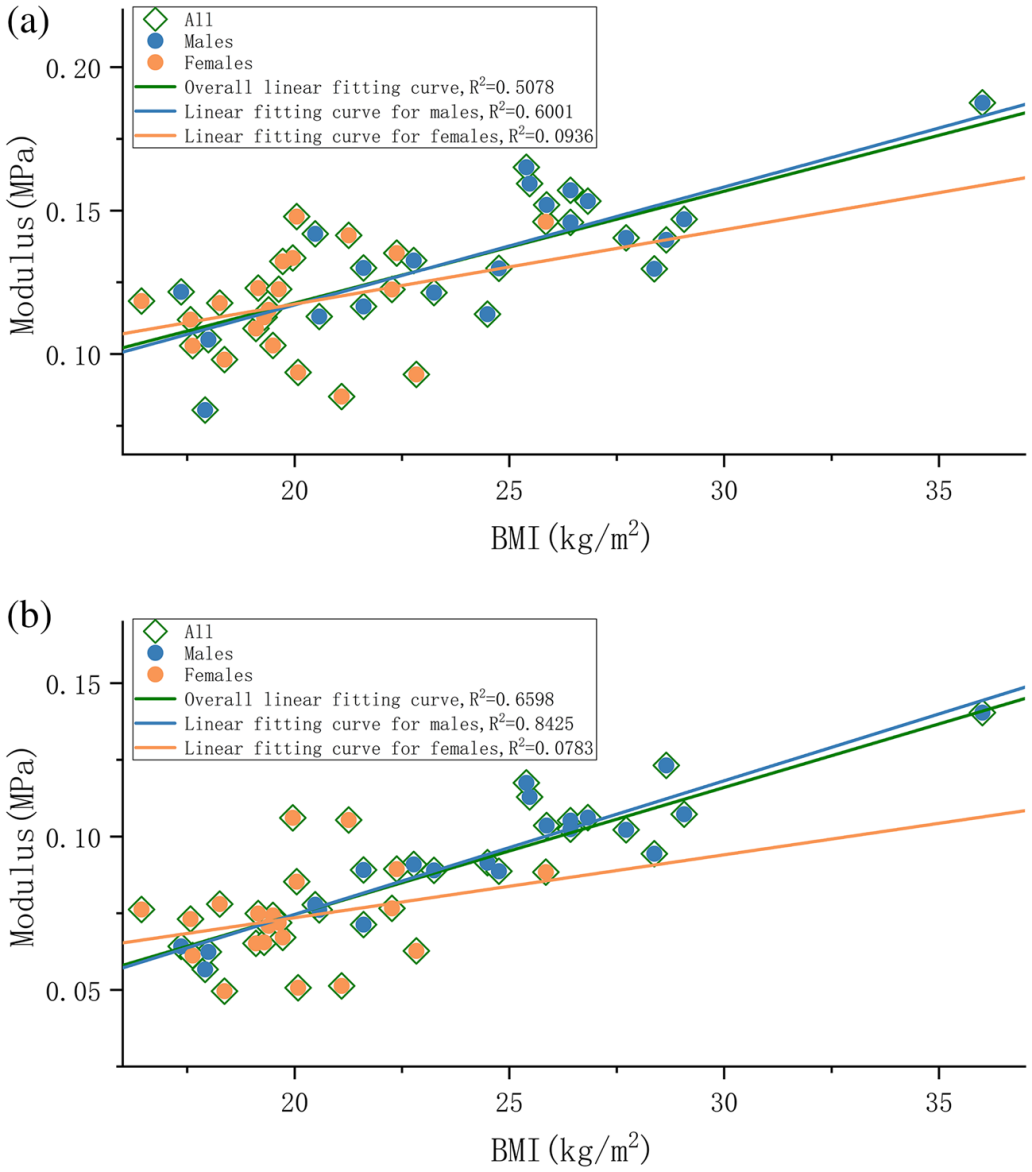
The distribution of moduli along with BMI, grouped by biological gender: The orange dots and trend line represent female, the gray dots and trend line represent male, and the blue trend line is for overall data: (**a**) The longitudinal shearing; (**b**) The transverse shearing.

The heatmaps illustrating the correlations (indicated by Spearman coefficient and along with P-value) in both shearing directions are presented in Fig. 5. First of all, we can observe that the main trends in either longitudinal or transverse are very similar, in this way we can discuss the correlations regardless of the shearing directions. In the green frame of the heatmap, we can find that the shear stress has a clear correlation to the BMI and weight, and BMI is more important than weight. Especially, the spots M2 and M5 show stronger correlation with the coefficients are 0.63/0.69 and 0.65/0.78 respectively, which agrees with our previous hypothesis that the upper-outer forefoot bears more shearing for the whole plantar^30,34^. Also, this observation on BMI or bodyweight correlation agrees with other’s study that claims higher body mass could cause changes in the mechanical properties of heel pad and plantar fascia^31^. The distributions of modulus along with BMI in two directions, grouped by biological gender are presented in Fig. 6.

For the discussion on gender difference, we have conducted Shapiro–Wilk tests to demonstrate that the height and BMI follow a normal distribution, whereas the age and weight do not. Variance ratio test revealed that the height conformed to variance homogeneity, whereas the BMI does not. Therefore, two-sample Wilcoxon rank-sum (Mann–Whitney) test was conducted on the age and weight data, two-sample t test with equal variances was conducted on the height, and two-sample t test with unequal variances was conducted on the BMI. The results indicate significant differences in weight (p < 0.0001), height (p < 0.0001), and BMI (p = 0.0001 < 0.05) between males and females, while there are no significant differences in age (p = 0.1437 > 0.05).

It is impressive that gender is highly negative correlated to the moduli, as a dark blue strip can be observed on Fig. 5. However, by noticing the correlation between gender and weight is − 0.77, it is natural to hypothesize that since usually female has lower weight than male, that here the gender difference is mainly the weight difference. In Fig. 6, from an intuitive perspective, the data distribution for different genders tends to be quite similar and adheres to a linear relationship where higher BMI corresponds to higher modulus. Nevertheless, the linear fit exhibits a low correlation (R value) due to the significant dispersion of data points. Furthermore, the R for the male dataset is significantly higher and very similar to the R for the entire dataset. This is likely due to the fact that the male subset contains more data points in the higher BMI range, which effectively extends the linearity of the overall distribution. This effect is particularly pronounced when there is an extreme data point with a high BMI at upper right, as it further elongates the overall region. However, the distribution range of female set is rather limited, which fails to effectively illustrate this linear relationship. To summarize, according to the available evidence, we can safely hypothesize that there is a positive correlation between BMI and shear modulus. The underlying mechanism is likely that those with a higher BMI are more likely to experience more stress in their daily lives, which in turn leads to greater exercise in their feet and an adaptation to a stronger modulus. Nonetheless, it is unclear that if gender difference is mainly about the weight difference, because currently the range of females’ weight is far shorter than male. At this point it cannot be concluded that gender is an effective variable, and we need more data covering a wider range of females’ bodyweight to further analyze in the future research.

The foot size exhibits a surprisingly weak link with the modulus, despite the intuitive expectation that size would significantly influence the foot's properties to bear everyday load. Better still, there is a notable correlation between width and spot L, with foot length having a lesser impact. This is logical since the load-bearing on the foot arch shall be more sensitive to width, comparing to the forefoot and heel. However, additional tests are necessary to achieve a more precise understanding.

Beside the moduli we defined in this manuscript, we may also compare the peak load achieved in strain to others’ reports on shear force experienced. The average shear stress we obtained is 46.3 kPa for all points using the formula Fs/A, and 38.5409 kPa for the forefoot. In previous studies, the peak maximal shear stress within the forefoot is measured between 18 and 158 kPa^15^. A study on plantar shear stress distributions reported peak shear on diabetic neuropathic group, diabetic control group, and healthy control group, which are respectively 91.3 kPa, 82.0 kPa and 64.6 kPa^37^. Perry et al. reported the greatest shear in the lateral metatarsal heads (33 kPa)^35^. Yavuz et al. discovered that the diabetic peripheral neuropathy (DPN) group had the highest regional peak shear at the hallux (77.9 kPa), which is higher than the non-DPN diabetic control (77.6 kPa,) and much more significantly than the healthy control groups (61.1 kPa,)^38^.

Last but not the least, the FEA simulation presents deviated results as seen in Table 1. The modulus at each anatomical site in either direction is larger than the overall range of reality test, but still comparable. Better still, the FEA demonstrates that the transverse moduli are consistently smaller than the longitudinal moduli. This finding provides support for the hypothesis that the directional variation in moduli is largely due to geometric factors. Nevertheless, the deviation implies the defects in FEA methodology or parameter setup, which should be further investigated in future research.

## Conclusions

We employed a novel in-vivo/in-situ stress–strain detection device, based on the principle of DMA, to study the human plantar soft tissue. This innovative device allowed us to access data on shear stress–strain behavior from five distinct locations on the plantar of 43 volunteers who participated in the study. The results were also compared with finite element analysis simulation. We unearthed some encouraging observations and established several hypotheses after thoroughly analyzing the obtained data: (1) the material properties of living people's soles, as well as soft tissue in different areas on the plantar, reveal considerable individual variances; (2) The moduli in the transverse direction are significantly smaller than in the longitudinal direction, which is easily comprehensible since the shear-bearing primarily occurs in the walking direction. Nevertheless, the correlation between moduli and other parameters in both directions is similar. (3) The higher modulus of M2 and M5 compared to M1 indicates that the outer portion of the forefoot is the more shear-bearing region; (4) It may safely hypothesize that BMI highly affects the shear modulus while the gender difference may also mainly due to the weights; (5) The foot size exhibits a surprisingly weak link with the modulus, better still, a correlation between width and spot L is still notable.

Although the data collection of 43 is relatively small and somehow unconvincing in this experiment, the conclusions and speculations drawn align with our expectations and daily experience. These intriguing findings provide crucial basic support for further in-depth investigations. There are still many exciting challenges to overcome in the future. We will invest in larger-scale data collection to acquire information from a more diverse demography, embracing differences in clinical characteristics. Subsequently, big data analyses will be employed to deeply explore the correlations.

By implementing the methodology presented in this study, we hope to gain comprehensive understanding of the incidence of plantar diseases and injuries in future research. This insight can be utilized as an early clinical monitoring and diagnostic tool, aid in the creation of individualized treatment and rehabilitation strategies, as well as the development of more efficient training approaches for professional athletes. Concurrently, investigating the distribution of shear stress in various regions of the plantar can assist in the development of footwear and supports that better conform to ergonomics. We hope that this work can make pioneering contributions to fields such as clinics, foot biomechanics, biomimetic materials, sports medicine, footwear, and foot care, while establishing a database to achieve a series of valuable applications.

### Supplementary Information

Supplementary Information.

Supplementary Information 1.

Supplementary Information 2.

## Data Availability

The main data generated or analysed to support the conclusion during this study are included in this published article and its supplementary information files. The full datasets generated and/or analysed during the current study are not publicly available due PRIVACY PROTECTION POLICY AND ETHIC REQUIERMENT but are available from the corresponding author on reasonable request.
