# Supplementary material for: An exploratory in-situ dynamic mechanical analysis on the shearing stress–strain mechanism of human plantar soft tissue
Source: Sci Rep. 2024 May 25;14:11953. doi: 10.1038/s41598-024-62713-9 (PMC11128022; doi:10.1038/s41598-024-62713-9)
Supplement: Supplementary file 2 — Supplementary Information 1. [file 41598_2024_62713_MOESM2_ESM.pdf]

Volunteer Testee Informed Consent Form for Physiological Health-related Information

Dear Volunteer Testee,

We are researchers from Fudan University in Shanghai, conducting a study as part of the National Key Research and Development Program: "Rehabilitation Mechanisms of Common Elderly Foot Diseases Database and Multi-System Coupling Effects" (Project No. 2022YFC2009501). We are conducting a study on "Biomechanical Properties of Plantar Soft Tissues" among healthy individuals across different age groups: young, middle-aged, and elderly. We are seeking your participation and input. In this regard, we will be conducting tests and collecting certain physiological data (biomechanical data of plantar soft tissues) from you for fundamental scientific research. We will need to measure some of your physical characteristics, such as height and weight, and record the biomechanical properties of different areas of your foot under various motion patterns, such as axial tension-compression, lateral shear, longitudinal shear, and torsion modes. We welcome volunteer testees aged between 18 and 65 years, without foot soft tissue or bone pain, functional impairments, and with sound physical and mental health. Both males and females are invited to volunteer testee. The test information will be stored at the Department of Biomedical Engineering Technology at the School of Engineering and Applied Sciences, Fudan University.

Additionally, your information may be stored for an extended period for the research program at Fudan University's "Human Genetic Resources Preservation and Management Center" (referred to as the Fudan Human Gene Database). This informed consent form will provide you with an understanding of this program, your rights as a volunteer testee, and other essential details. We urge you to carefully read the following content. If you have any questions or find certain aspects unclear, please inquire with the project research representative (researchers, investigators, or staff). Your signature on this informed consent form is only valid when you have fully understood the answers to your questions and willingly choose to be volunteer testee in this study.

1. Purpose and Significance of Information Collection

The purpose of collecting and retaining your information is primarily to establish a method for in vivo characterization of human plantar soft tissue biomechanical properties and to assess the

variations in these properties across different populations. The data collection process will adhere strictly to designated protocols and requirements. Simultaneously, the utilization of this data in the current project and future research endeavours will contribute to the advancement of medical research in our country and enhance the overall health and well-being of the population.

2. Use of Information

We collect your information and data to complete scientific research projects entrusted by the national or local government, and the purpose is public welfare and non-profit. Your biological data and information are used in a wide range of medical and health science research in order to further the public interest to the greatest extent possible, and may also be used for as yet unknown research purposes. Nor can we describe in detail all possible future medical research projects. We will not conduct activities that violate laws and regulations, ethics and national interests with your information and data.

Data retention for teaching and research will be long-term. Information and data may be provided to domestic scientific research institutions other than Fudan University for cooperative health science research, and may also be provided to foreign scientific research institutions for international cooperative research. Under the premise of complying with the national genetic resources management regulations and sharing agreements, any All use must go through the strict review process of the Ethics Committee of Fudan University to protect your rights and interests. All relevant opinions of the Ethics Committee will comply with the requirements of relevant national laws and regulations on human genetic resources.

3. Discomfort, Risks, and Protective Measures Related to Biological Data Volunteer testees

We will assign a unique identifier to you and create a file containing information such as gender, height, and weight. Research personnel will guide you in placing your feet in designated positions on the biomechanical testing apparatus for plantar soft tissue. You will be asked to position five areas of your foot – the left, middle, and right foot areas, arch, and sole – in specified locations. This involves completing four deformation modes: axial tension-compression, lateral shear, longitudinal shear, and torsion. Each mode will be tested three times, resulting in a total of 60 sets of valid data. The process of collecting data on plantar biomechanical properties will not affect your physical health. While the collection process may cause some discomfort in the soles of your feet, you can communicate any discomfort to the research personnel during the experiment. The research team will promptly assess and terminate any potential harmful factors. If you experience

harm due to the collection procedure, you are entitled to receive treatment and compensation as stipulated by law.

4. Benefits and Significance for Volunteer testees

Being volunteer testees in this study means that you will not get financial benefits from it, and you will most likely not get any financial and medical benefits from the research results. But future research results may benefit society, and even your children and grandchildren. If the research results derive any intellectual property rights such as patents or other transformation results, because the research results are derived from the research and analysis results of the deprived volunteer testee groups, all rights and interests will not be directly related to you personally.

Your information collection cannot directly get financial rewards, and the health tests and various scientific experiments during your participation in the research are free of charge, and you do not need to bear additional costs. Of course, you have spent precious time by cooperating with scientific research and testing, we will pay you a reasonable labour and transportation subsidies as a gratitude.

Due to the relatively short time for scientific research projects, it is impossible to predict whether the results of the research will help and guide the health of you or your family members. If a major health problem is discovered about you during the study, we will respect your decision whether to provide this information to you and/or your family.

5. Confidentiality and Measures for Volunteer testees' Personal Information

Various health science-related research conducted by Fudan University does not involve your personal privacy and identifiable information. Fudan Human Remains Database is obliged to strictly protect the privacy and personal information of each volunteer testee, and adopts the following protection methods: (1) Establish confidentiality measures and a volunteer testee information security system (personal information protection system), and all collected Data is managed anonymously (code management); (2) securely store data and data, and set access rights to all data and/or data in the database; (3) when data or prepared research materials are provided (data for scientific research purposes) When transferring) to other researchers or institutions, no research institution or individual will obtain your personal identity and privacy information; (4) The data obtained from the research may be published or announced publicly, but your name or identifiable identity will not be announced Personal data; most of the data is used for basic research, but a small part may be given to indirect commercial purposes, such as the development of new drugs or the

screening of biomarkers. You have the right to decide whether to allow anonymized data information to be used for commercial purposes.

6. Volunteer testees Right to Autonomous Decision

You have the right to refuse to sign this informed consent, and refusing to sign this informed consent will not affect any of your rights.

You can withdraw unconditionally at any time, that is, request to withdraw from the test. According to the agreed content, the Institute of Biomedical Engineering Technology, Fudan University Engineering and Applied Technology Research Institute will no longer collect or use or store biological data from you. You can always contact the staff who informed you of the information at that time [Dr. Ran Huang, Tel: 15001979958]. At that time, you will be required to sign a withdrawal statement. According to your withdrawal request, Fudan Human Remains Database will destroy the identifiable biological data from you, and will not continue to collect and/or use it. The Fudan Human Relics Database will keep the destruction records for a period of time for inquiries.

7. Others

If you have any other questions about participating in this research, you can always contact the scientific researcher [Dr. Ran Huang, Tel: 15001979958] who informed you of the information at that time. We will promptly answer any questions you may have. Thank you for your interest in our research projects, whether you are involved or not.

Informed Consent Statement

1. I have read and understood the entire content of this informed consent form.
2. I had the opportunity to ask questions and all questions were answered.
3. I understand that my participation in this activity is entirely voluntary. I can also choose to withdraw from this activity at any time.
4. I □**wish** □**do not wish** to be informed about any research results that have significant relevance to my health.
5. I □**wish** □**do not wish** for my anonymized data or information to be used for commercial purposes.
6. I understand that after signing, I can still seek clarification from the personnel of Fudan University's Ethics Committee.
7. I agree to provide trial data and information to Fudan University's scientific researchers for the completion of this national scientific research program; [ ]
8. I agree to provide trial data and information to Fudan University's scientific researchers for the completion of this and all future national-level scientific research programs, and I also agree to share research data with domestic and foreign research institutions. I authorize the Ethics Committee of the School of Life Sciences at Fudan University to review the appropriateness of using my data and information; [ ]

Volunteer testee Signature: Date: Year Month Day

(Note: If the volunteer testee is unable to write their name, the signature and date must be provided by the legal agent)

Legal Agent Signature: (Relationship with patient:)

Date: Year Month Day

Fudan University Informant Declaration

I have accurately explained the entire content of the informed consent form to the volunteer testee, answered all questions raised by him, and provided him with a signed copy of the informed consent form.

Signature of Fudan University informant: Ran Huang Date: Year Month Day

生理健康相关信息受试者知情同意书

亲爱的受试者：

我们是上海复旦大学的科研工作者，为完成国家重点研发计划(主动健康和老龄化科技应对)：《老年常见足病数据库及多系统耦合作用的康复机制研究》2022YFC2009501，我们将在健康青、中、老年人群中开展“足底软组织生物力学性能检测”的研究。在此，我们特向您征求意见。我们将测试并收集您的一些生理数据（足底软组织生物力学数据），用于基础科学研究。我们需要对您的体质特征做一个测量，如身高和体重等，并且记录您足底不同部位在不同运动模式下的生物力学性能，如轴向拉压模式、横向剪切模式、纵向剪切模式和扭转模式等。我们将接受年龄 18 周岁-65 周岁，无足底软组织、骨骼疼痛以及功能障碍，且体貌和精神健康人员的数据受试，男女不限。测试信息将保存在复旦大学工程与应用技术研究院生物医学工程技术研究所。此外，您的信息也可能长期保存用于复旦大学“人类遗传资源保藏管理中心”（简称复旦人遗数据库）的科研计划。本知情同意书将告诉您这项计划，以及您作为受试者的权利等重要信息，请认真阅读下列内容，如果您有任何问题或是对某些内容不理解的话，请询问项目研究代表（研究人员、调查员或者工作人员），只有当您对这些问题的解答充分理解，并自愿参加本研究时，方可签署该知情同意书。

一、收集信息的目的和意义

收集并保存您的信息，本研究主要目的是建立一种活体表征人体足底软组织生物力学性能的方法，并检测不同人群中人体足底软组织生物力学性能的差异。数据的采集将严格按照规定流程和要求完成。同时，这些数据在本项目和今后研究中使用，有助于促进我国医学发展，提高人民健康水平。

二、 信息的用途

我们收集您的信息和数据是为了完成国家或地方政府委托的科研项目，其目的是公益性的、非盈利性的。为了最大可能地促进公共利益，您的生物数据和信息会用于广泛的医学与健康科学研究中，也可能应用于目前还未知的研究目的。目前我们也不能详细描述出所有将来可能出现的医学研究项目。我们不会对您的信息和数据进行违反法律法规、伦理道德和国家利益的活动。

—

用于教学和研究的数据保存将是长期的。信息和数据可能提供给复旦大学以外的国内科研机构使用，用于合作开展健康科学研究，也可能提供给国外科研机构开展国际合作研究，在符合国家遗传资源管理条例和共享协议的前提下，任何使用均需经过复旦大学伦理委员会严格的审查程序来保障您的权益，伦理委员会所有相关意见均会符合国家相关人类遗传资源法律法规的要求。

三、生物数据受试可能给受试者带来的不适、风险和保护措施

我们将对您进行编号，建立包含性别、身高和体重等信息的档案。将由研究人员指导您将脚放置在足底软组织生物力学性能试验样机的指定位置。研究人员将要求您将脚掌（左、中、右）、足弓以及足底等 5 个部位放于指定位置，完成轴向拉压、横向剪切、纵向剪切、扭转等 4 个形变模式，各模式须重复测试 3 次，共需 60 组有效数据。我们采集足底生物力学性能的过程，不会影响您的身体健康。采集过程中，可能会造成足底不适。您可以在试验过程中向研究人员反馈您的不适。研究人员将即时评估并及时终止可能的损害因素。如因采集行为导致您受到损害，您将依法获得治疗和赔偿。

四、受试者的益处和意义

受试意味着无法从中获得经济利益，您个人极有可能不会获得任何研究成果带来的经济和医疗上的收益。但未来研究成果可能会造福于造福社会，乃至惠及您子孙后代。研究结果若衍生任何知识产权如专利或其他转化成果时，因为研究结果都源于去隐私化的受试者群体的研究分析结果，所以所有权益都将与您个人没有直接关联。

您的信息采集都不能直接获得经济报酬，您参与研究过程中进行的健康检测和各种科学实验都是免费的，不需要您额外承担费用。当然，您配合科学研究和检测，您耽误了宝贵的时间，应得到一些务工补贴；以及参与研究往返实验场所所需的交通补贴。

由于科研项目的开展时间相对不足，因此也无法预知研究所得的结果是否对您或您的家属健康有所帮助和指导。若在研究中发现有关您的重大健康问题，我们将尊重您的决定而选择是否将此信息提供给您本人和/或家属。

五、受试者个人资料的保密范围和措施

复旦大学从事的各类健康科学相关研究，不涉及您的个人隐私和可识别信息。复旦人遗数据库有义务严格保护每一位受试者的隐私和个人信息，并采取以下保护方式：（1）建立保密措施和受试者信息安全制度（个人信息保护制度），对所有采集的数据采取匿名化管理（编

—

码管理）；（2）安全贮存数据和数据，对在库所有数据和/或数据设置访问权限；（3）当数据或制备的研究材料提供（以科研为目的的数据转移）给其他研究者或机构时，任何研究机构或个人都不会获得您的个人身份和隐私信息；（4）研究所得数据可能公开发表或公布，但不会公布您的姓名或可辨识身份的个人资料；数据绝大多数情况用于基础研究，但也有少部分可能被赋予非直接的商业用途，如用于新药的研发或生物标志物的筛选。您有权决定是否允许匿名化的数据信息用于商业用途。

六、受试者的自主决定权

您有权拒绝签署本知情同意书，拒绝签署本知情同意书不会影响您的任何权益。

您可随时无条件退出，即要求撤销受试，按照约定的内容，复旦大学工程与应用技术研究院生物医学工程技术研究所将不再采集或利用或保藏来源于您的生物数据。您可以随时联系当时向您告知信息的工作人员[黄然老师，电话：15001979958]。届时需要您签署一份退出声明，根据您退出的要求，复旦人遗数据库将对来源于您的可辨识的生物数据进行销毁、不再继续采集和/或利用。复旦人遗数据库将在一段时间内保留销毁记录以备查询。

七、其他

如果您有其他关于参与本研究的任何问题，您可以随时联系当时向您告知信息的科研人员 [黄然老师，电话：15001979958]。我们将及时答复您的任何问题。无论您是否参与，都感谢您关心我们的研究项目。

—

知情同意声明

1. 我已经阅读并理解了本知情同意书的全部内容。
2. 我有机会提问而且所有问题均已得到解答。
3. 我理解参加本活动完全是自愿的。我也可以选择在任何时候退出这一活动。
4. 我 **☐希望 ☐不希望** 知道任何与我的健康有重大关联的研究结果。
5. 我 **☐希望 ☐不希望** 自己匿名化的数据或信息用于商业用途。
6. 我清楚签署以后还有疑问可以咨询复旦大学的伦理委员会的工作人员。
7. 我同意受试数据和信息给复旦大学的科技工作者，以供完成本次国家科学研究计划;【 】
8. 我同意受试数据和信息给复旦大学的科技工作者，以供完成本次及今后的所有国家级科学研究计划，并且同意国内外的科研机构共享研究数据，并授权复旦大学生命科学学院伦理委员会审核使用本人数据和信息的适当性。【 】

受试者签名： 日期： 年 月 日

（注：如果受试者无法书写名字时，则需代理人签名和签署日期）

代理人签字： （与患者关系 ）

日期： 年 月 日

复旦大学告知信息者声明

我已经准确地向受试者解释了知情同意书的全部内容，回答了其所提出的所有问题，并提供其一份签署过的知情同意书副本。

复旦大学告知信息者签名：[signature] 日期： 年 月 日
