# Supplementary material for: An exploratory in-situ dynamic mechanical analysis on the shearing stress–strain mechanism of human plantar soft tissue
Source: Sci Rep. 2024 May 25;14:11953. doi: 10.1038/s41598-024-62713-9 (PMC11128022; doi:10.1038/s41598-024-62713-9)
Supplement: Supplementary file 3 — Supplementary Information 2. [file 41598_2024_62713_MOESM3_ESM.pdf]

HIRB Approval Letter

Statement: HIRB is composed and works in accordance with China GCP and relevant regulations, and its review and work process will not be affected by any organization or individual other than HIRB.

Approval No.: (2022) Temporary Trial No.(889)

Review Meeting Date	2022/9/27
Review Meeting Venue	Tencent video meeting
Research Title	The Database of Common Foot Diseases in the Elderly and Research on Rehabilitation Mechanism of Multi-system Coupling
Review Documents	Attached list of ethical review documents (stamped page)
Sponsor	None
Clinical Research Institution	Department of Orthopedics, Huashan Hospital, Fudan University
Principal investigator	Ma, Xin
Ethics review methods	■ Meeting Review ☐ Quick Review ☐ Emergency Meeting Review
Ethic Committee members	Wu, Rong (Researcher, female), Gu, Yong (Professor of medicine), Ni, Quanxing (Professor of medicine), Feng, Xiaoyuan (Professor of medicine), Wang Dayou (Chief Pharmacist), Dong Qiang (Professor of medicine), Yang, Qinping (Professor of medicine, female), Wang, Jian (Professor of medicine), Ding, Ding (Associate researcher, female), Cao, Guoying (Deputy Chief Pharmacist, female), Chen, Jianbo (Lawyer, other affiliation), Wang, Yuejuan (Non-medical major, female, other affiliation), Liu, Haitao (Ethics, female, other affiliation), Zhong, Liang (Professor of medicine), Huang, Haihui (Professor of medicine, female), Wu, Cuiyun (Assistant researcher, female)
Review opinion	1. Ethical Review Committee's Review: Agree to conduct the research according to the amendments.<br>Comments and Suggestions: None<br>2. Annual/Periodic Review of the research implementation process by the Ethical Review Committee: ■ Yes ☐ No<br>Review frequency from the date of initial approval:<br>☐ 3 months ☐ 6 months ■ 1 year<br>3. The Ethical Review Committee has the right to change the annual/periodic review frequency based on the actual progress.

Committee Chairman or vice Chairman Signature:

Ethical Review Committee of Huashan Hospital, Fudan University (official seal)

复旦大学附属华山医院 伦理审查委员会 HIRB

Date:

Attention: (Please read carefully)

1. All matters related to the protection of human genetic resources or requiring special approval from relevant departments according to national regulations must be declared to and approved by the relevant departments before project execution.
2. Approved projects by this Ethical Review Committee are all related to biomedical research involving humans and must strictly adhere to the approved protocols in terms of timelines and participant numbers. Any changes must be approved by the Hospital Medical Ethics Committee and the relevant departments for clinical trials.
3. This approval letter may be filed with the central institutions and their ethics committees. Contact the Ethical Review Committee for any disagreements regarding protocol feasibility (including qualifications and experience of the researchers, equipment and conditions, etc.)

4. Research information should be registered on publicly accessible websites, such as China's Medical Research Registration and Filing Information System, before the enrollment of the first participant.
5. Approved projects must adhere to the protocols approved by the Ethical Review Committee and comply with ethical principles defined by national regulations, NMPA guidelines, and the Helsinki Declaration.
6. Please inform the Ethical Review Committee promptly of any suspension or early termination of clinical research.
7. Please report any suspected and unexpected serious adverse reactions to the Ethical Review Committee promptly.
8. Any modifications to approved clinical research protocols, informed consent forms, or changes in the principal investigator, must be submitted to the Ethical Review Committee in a timely manner and can only be implemented after obtaining approval from the committee. Minor administrative changes that only involve clinical trial management, such as the replacement of monitors or telephone numbers, may be exempted but should be reported promptly.
9. Report instances of non-compliance/deviation from the protocol that increase participant risks or significantly affect the study implementation, or to avoid immediate harm to participants. Other instances of general non-compliance/deviation from the protocol may be reported periodically.
10. Submit an application for review one month prior to the due date of the annual/periodic review, regardless of whether the study has commenced or not, based on the Ethical Review Committee's opinions.
11. Submit a final report to the Ethical Review Committee upon completion of the clinical research.

Address: No.12, Wulumuqi Middle Road, Shanghai Post code: 200040 Tel: 021-52888045

伦理审查同意函

HIRB Approval Letter

声明：本伦理审查委员会按照中国 GCP 和有关法规组成和工作，其审查和工作过程不受伦理审查委员会以外任何组织及个人的影响

批件号：（2022）临审第（889）号

审查会议日期	2022 年 9 月 27 日
审查会议地点	腾讯视频会议
研究项目名称	老年常见足病数据库及多系统耦合作用的康复机制研究
审查文件	随附伦理审查文件清单（盖章页）
申办者	无
临床研究单位	复旦大学附属华山医院骨科
主要研究者	马昕
伦理审查方式	■ 会议审查　口快速审查　口紧急会议审查
审查委员	伍蓉（研究员，女），顾勇（医学教授），倪泉兴（医学教授），冯晓源（医学教授），王大猷（主任药师），董强（医学教授），杨勤萍（医学教授、女）、王坚（医学教授），丁玎（副研究员、女），曹国英（副主任药师，女），陈建波（律师，外单位），王跃娟（非医药专业，女，外单位），刘海涛（伦理学，女，外单位），钟良（医学教授），黄海辉（医学教授，女），吴翠云（助理研究员，女）
审查意见	1. 经本伦理审查委员会审查：同意按修正案进行研究<br>意见和建议：无<br>2. 伦理审查委员会对该研究实施过程的年度/定期审查：■是　口否<br>审查频度为研究首次同意之日起：　口 3 个月　口 6 个月　■1 年<br>3. 伦理审查委员会有权根据实际进展情况改变年度/定期审查频度。

主任委员或副主任委员签字：[illegible]

复旦大学附属华山医院伦理审查委员会（盖章）

日期：2022.11.28

注意：（请仔细阅读）

1. 凡是涉及人类遗传资源保护或者按照国家规定必须经有关部门专项审批的内容，均必须在项目执行前向有关部门申报并获得同意。
2. 本伦理审查委员会同意的项目均为涉及人的生物医学研究，必须严格按照所同意方案规定的期限和受试者例数完成，不得随意超过。如需作为临床项目常规开展，必须经医院医学伦理委员会同意，并按照国家新技术申报相关规定向有关部门申请同意。
3. 本同意函可能在各中心机构及其伦理委员会备案。如果对方案在贵机构的可行性（包括研究者的资格与经验、设备与条件等）有不同意见，请及时与本伦理审查委员会联系。
4. 在第 1 例受试者入组之前，研究信息应在公众所及的网站上登记，如我国医学研究登记备案信息系统。
5. 已同意项目须遵循本伦理审查委员会同意的方案执行，须符合国家各部委、NMPA 相关法规指南和《赫尔辛基宣言》等我国认可的国际指南规定的伦理原则。
6. 暂停/提前终止临床研究，请及时通知伦理审查委员会。
7. 发生可疑且非预期的严重不良反应，须经研究者快速报告本伦理审查委员会。
8. 对已获得伦理审查同意的临床研究方案、知情同意书等材料的修改及主要研究者更换等，须及时递交本伦理审查委员会，获得审查同意后方可执行，但为了及时消除对受试者的紧急危害（事后及时报告伦理审查委员会）或更换监查员、电话号码等仅涉及临床试验管理方面的改动除外。
9. 发生增加受试者风险或显著影响研究实施的不依从/偏离方案事件，或为了避免对受试者的即刻危险偏离方案，应及时报告本伦理审查委员会；其他一般不依从/偏离方案事件可定期报告。
10. 根据伦理审查委员会对年度/定期审查频率的意见，无论研究开始与否，请在年度/定期审查日到期前 1 个月提出审查申请。
11. 完成临床研究，须提交结题报告给本伦理审查委员会。

地址：上海市乌鲁木齐中路 12 号；邮编：200040；电话：021-52888045
